# Supplementary material for: Blood-Vessel-Inspired Hierarchical Trilayer Scaffolds: PCL/Gelatin-Driven Protein Adsorption and Cellular Interaction
Source: Polymers (Basel). 2022 May 24;14(11):2135. doi: 10.3390/polym14112135 (PMC9182901; doi:10.3390/polym14112135)
Supplement: Supplementary file 1 [file polymers-14-02135-s001.zip › polymers-1710565-supplementary.pdf]

Supplementary Material

Multilayered scaffolds from biodegradable PCL: Tunable bioactive and mechanical properties

*Maria A. Rodriguez-Soto, Andres J. Garcia-Brand, Alejandra Riveros, Natalia Suarez V, Fidel Serrano, Johann F. Osma, Carolina Muñoz-Camargo, Juan C. Cruz, Nestor Sandoval and Juan C. Briceño **

Supplementary Methodology

File S1. Crystallinity calculation from DSC thermographs

Glass transition (T_g), crystallization (T_c), and melting (T_m) temperatures were determined from the second heating, while the apparent crystallinity (X_c) was calculated by Equation (S1) as representative thermal parameters.

$$X_c(\%) = \frac{\Delta H_m^{obs}}{\Delta H_m^0} \quad \text{(S1)}$$

Where ΔH_m^{obs} is the observed heat of fusion for each sample and ΔH_m^0 is the heat of fusion for 100% crystalline PCL homopolymer (ΔH_m^0 = 163 J/g).

File S2. X-Ray diffraction analysis

PCL and O-PCL X-ray diffraction analysis (XRD) were taken in a Rigaku Ultima III X-ray diffractomer (Tokio, Japan) in the Bragg-Brentano configuration. Samples with an average contact area of 25 mm^2 were placed into a bounding grid of 5 mm and exposed to a Bragg difraction angle (2θ) sweep between 5° and 50° with a 1.5°/seg step using Kα radiation with a Copper (Cu) anode of 40 kV and 40 mA.

The Debye-Sherrer equation (Equation (S2)) was used to estimate the apparent crystal size (D) of the polymeric structure based on the full-width at half-maximum (β) of the x-ray diffraction line, also known as FWHM, the wavelength of the X-ray used (λ) which was 1.5406 nm and the angle between the incident ray and the scattering planes (θ). In addition, the constant 0.9 was used in Equation (S2) to include the degree of crystal symmetry of pure PCL previosly reported by Doyle, S.E. et al. [77]

$$D = 0.9\frac{\lambda}{\beta\cos\theta} \quad \text{(S2)}$$

The phase content of each sample was calculated from the XRD profiles by peak deconvolution using the Materials Data Jade 9® Software (Newtown Square, PA, USA) using Gaussian profiles as fitting methods. Brifely, the crystaline and amorphous phases on the diffraction pattern were determined by fixing the halo peak at 20° and then establishing the best fit of the (100), (111) and (200) orthorhombic crystalline phase peaks. Finally, the crystalinity (x_c) of each sample was calculated according to Equation (S3).

$$x_c = \frac{I_c}{I_a + I_c} \quad \text{(S3)}$$

Where I_c represents the total crystalline area bellow the deconvoluted peaks with a maximum near to 21.4°, 22° and 23.5° and I_a the amorphous area under the deconvoluted halo with a maximum at 21°.

File S3. First Layer Mold Fabrication

For the mold fabrication (Supplementary Fig. S4), a thin layer of MS 319 UV-sensitive photoresist resin was deposited along a rectangular glass (25.4 x 76.2 mm) by spin-coating at 500 RPM for 20 seconds, followed by a thermosetting at 105°C to consolidate and harden the photoresist. Then, the glass containing the resin was photopatterned in a microgravure system SF 100 (ZKTeco, Dongguan, China) using an AutoCAD® designed photopositive mask printed with Helium-Neon laser (NHS) in a photoplotter Filmstar-Plus, using AGFA G333c and AGFA G101c at 2:1 volume ratio in water as media and fixed solutions, respectively, for 1 min each.
Therefore, the silicon was directly patterned by positioning the mask on top of the glass slide containing the harden silicon and exposed to UV-light (120 mJ/cm2) for 20 seconds with a KARL SUSS MJB3 UV400 aligner to weaken the regions not protected by the mask. Finally, the glass slide was immersed in a commercial SC 1827 Photoresist developer (MicroChem, Newton, MA) for 1 min, hardened at 105°C for another minute and etched by chemical attack with hydrofluoric acid to remove the weakened material. Next, the slides were passed through a PRS 1000 stripper (Avantor, Radnor Township, PA, USA) for 20 seconds to remove the remains of the photoresist.

File S4. Fiber Alignment Analysis by SEM

In order to evaluate the relative fiber aligment of the electrospun layers, a Fast Fourier Transform (FFT) analysis of SEM images at 500X was performed using ImageJ®. For the analysis, uncompressed TIF files containing 8-bit images of 1024x1024 pixels were processed with the FFT tool to translate optical information into the frecuency domain represented as a grayscale image with pixels distributed in a pattern that reflects the degree of fiber aligment. Then, the oval profile plugin (authored by Bill O'Connell) was used to extract radial summing of the pixel intensities for each angle between 0° and 360° in an oval projection forming a circle discretized into 100 points. The obtained values were normalized by the minimum value in the dataset and plotted in arbitrary units ranging from 0 to 0.15. Peaks height of the most prominent peaks every 90° were used as reference for fiber aligment analysis since the degree of alignment present in the original image is reflected by the height and overall shape of the most prominent peaks of the alignment plot. Finally, the 2D FFT aligment value was extracted as the peak value at 90° and values greater than 0.065 units (>0.065) together with two larger peaks at 90° and 270° were interpreted as aligned fibers.

Supplementary Material.

PCL oxidation process

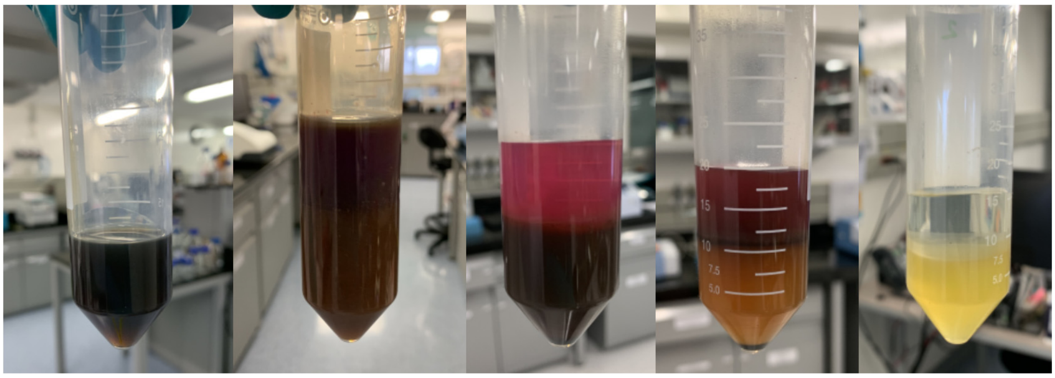

Figure S1. PCL oxidation process and several wash steps to purify the polymer.

Calibration curve of platelet activity assay

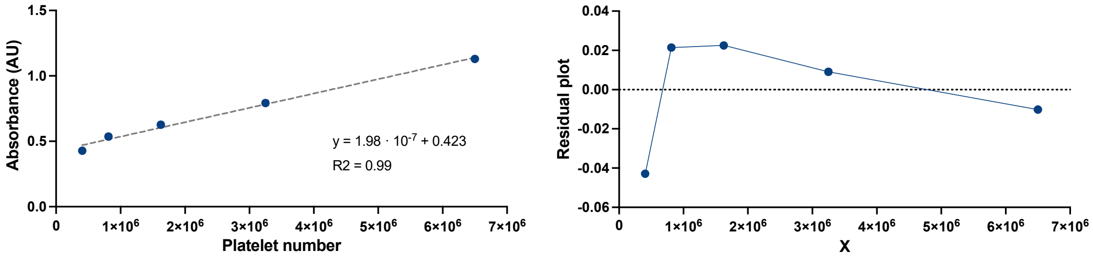

Figure S2. Calibration curve of platelet activity assay by lactate dehydrogenase (LDH) release.

Table S1. Simple linear regression data for platelet number quantification via LDH release.

Item	Best-fit Value	Std. Error	95% Confidence Intervals	
			Lower Value	Upper Value
Slope	1.98e-7	6.379e-9	8.953e-8	1,301e-7
y-intercept	0.4263	0.0214	0.3582	0.4944
x-intercept	-3881595	-	-5401961	2814091
**Goodness of Fit**				
R Squared	0.99			
Sy.x	0.0316			
**Slope significancy non-zero**				
F	296.4			
p-value	0.0004			
Deviation from zero	Significant			

X-Ray Diffraction Measurements

Fig. S3 shows the XRD pattern of PCL and O-PCL films at room temperature. XRD pattern of both samples displays three strong diffraction peaks at Bragg angles 2θ = 21.4°, 22° and 23.8° that correspond to the orthorhombic planes (110), (111) and (200) of the crystalline phase of PCL [41]. Also, a halo centered near 21° in the XRD profile indicates the presence of amorphous structures in the samples as expected from the semicrystalline structure of PCL [78]. Observable differences can be identified in the deconvoluted peak intensities and peak widths among the orthorhombic unit cells diffractions when oxidation takes place, indicating variations on the lamellar organization of the polymer [79].

Crystallinity changes explored by the average crystal size calculated with the Scherrer's formula and the crystallinity percentage changes upon oxidation are shown in Table 3. The calculated crystal size of each orthorhombic crystal lattice and the degree of crystallinity showed an average decrease of 13% and 12%, respectively, which agree well with crystallinity changes estimated from the FTIR spectra. Moreover, the reduced crystallite sizes of O-PCL have been correlated with a molecular weight loss most likely induced by degradation during the oxidation process [80]. The reduced crystallinity indicates a less ordered structure possibly caused by an early chain rearrangement and lamellar thickening assisted by the increase on inter-chain H-bonding in the functionalized polymer [41], [78].

Nevertheless, since there were no additional peaks on the XRD pattern of the O-PCL, it can be inferred that the oxidation process did not significantly affect the semicrystalline structure and thus, only appears to be a surface phenomenon [79]. Finally, the XRD pattern exhibits that are comparable with those obtained in previous studies where OCFG on the backbone of PCL was generated with CO_2 plasma treatment, indicating that the oxidation introduced here is a cost-effective alternative for polyester oxidation [78], [80].

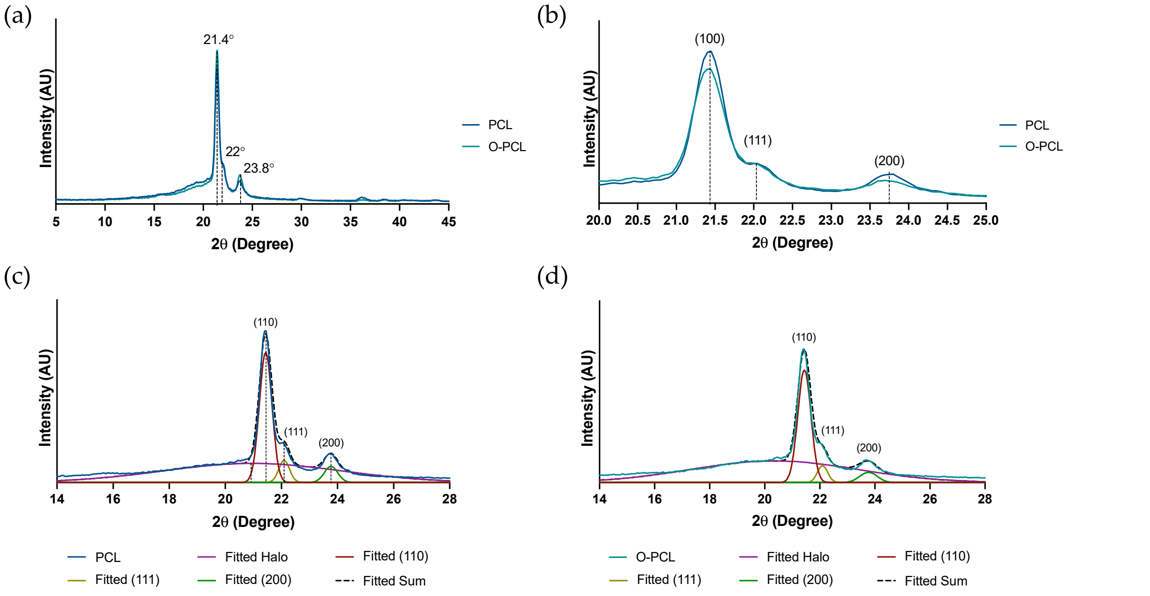

Figure S3. XRD patterns of PCL and O-PCL acquired from 5 to 45°. (a) XRD profiles of neat PCL and O-PCL. Characteristic Bragg-diffraction angles for the crystal structures of PCL are highlighted. (b) Magnification of the (110), (111) and (200) orthorhombic planes in the XRD pattern. Deconvoluted XRD pattern of the (c) PCL and (d) O-PCL, showing Gaussian fittings of (110), (111) and (200) crystal phases and the amorphous halo. AU stands for arbitrary units.

Table S2. Crystallinity and crystal size changes between PCL and O-PCL.

Sample	Crystal Size (Å)				X_c (%)
	(110)	(111)	(200)	Average	
PCL	1.48	1.29	1.89	1.56	38.04
O-PCL	1.35	0.99	1.78	1.37	33.63

Microgrooved mold fabrication

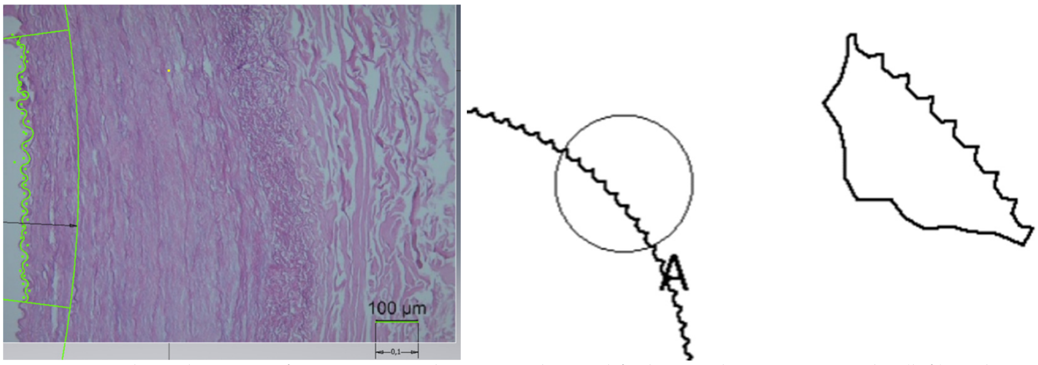

Figure S4. Histological section of porcine carotid artery with simplified intimal microtopography (left) and reconstructed pattern for the mold fabrication (right).

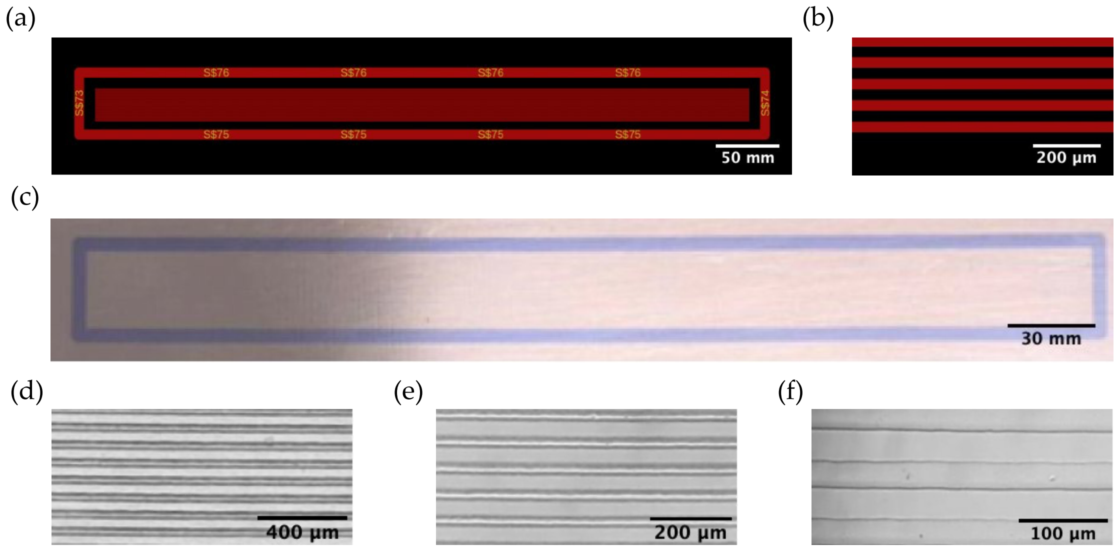

Figure S5. Fabrication of the glass mold. (a) Gerber file for the fabrication of the HNS physical mask. (b) Magnification of the Gerber file. (c) HNS printed physical mask. Glass mold with the microogrove pattern at magnifications of (d) 10X, (e) 20X and (f) 40X.

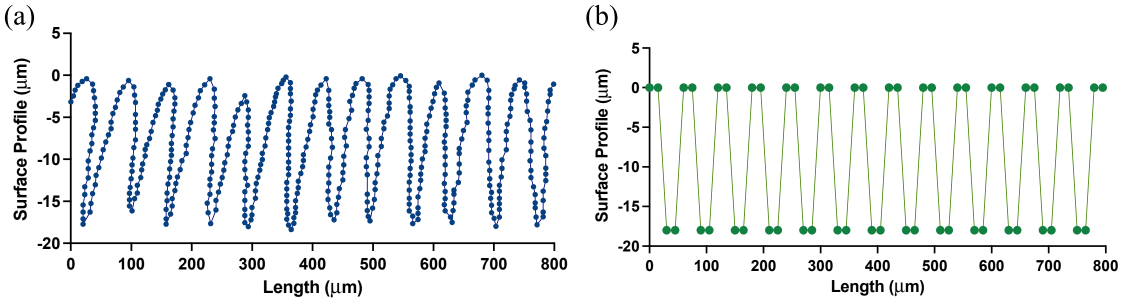

Figure S6. (a) Representative profilometer measurements of the microgrooved channels imprinted on the O-PCL film. (b) Estimated error based on expected values for an ideal imprinted morphology as measured with a probe at equidistant points along the sample length. An error of 16.28% ± 8% was estimated from 20 randomly selected probe points.

Bovine Serum Albumin (BSA) Calibration curve

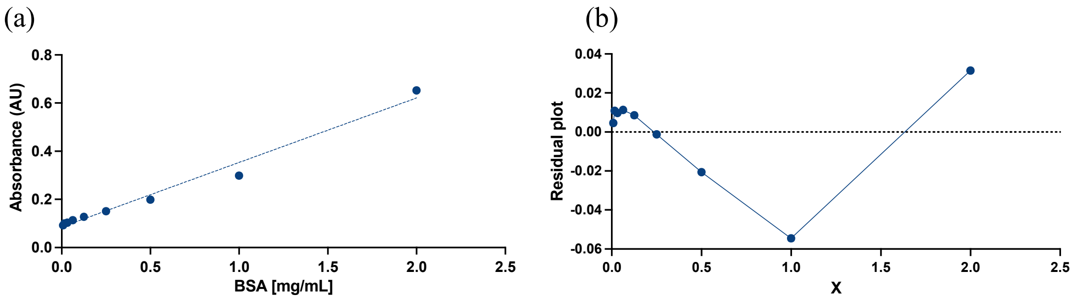

Figure S7. BSA Calibration curve for protein quantification by BCA assay. (a) Absorbance according of BSA amount. (b) Residual plot.

Isotherm and kinetics models fitting

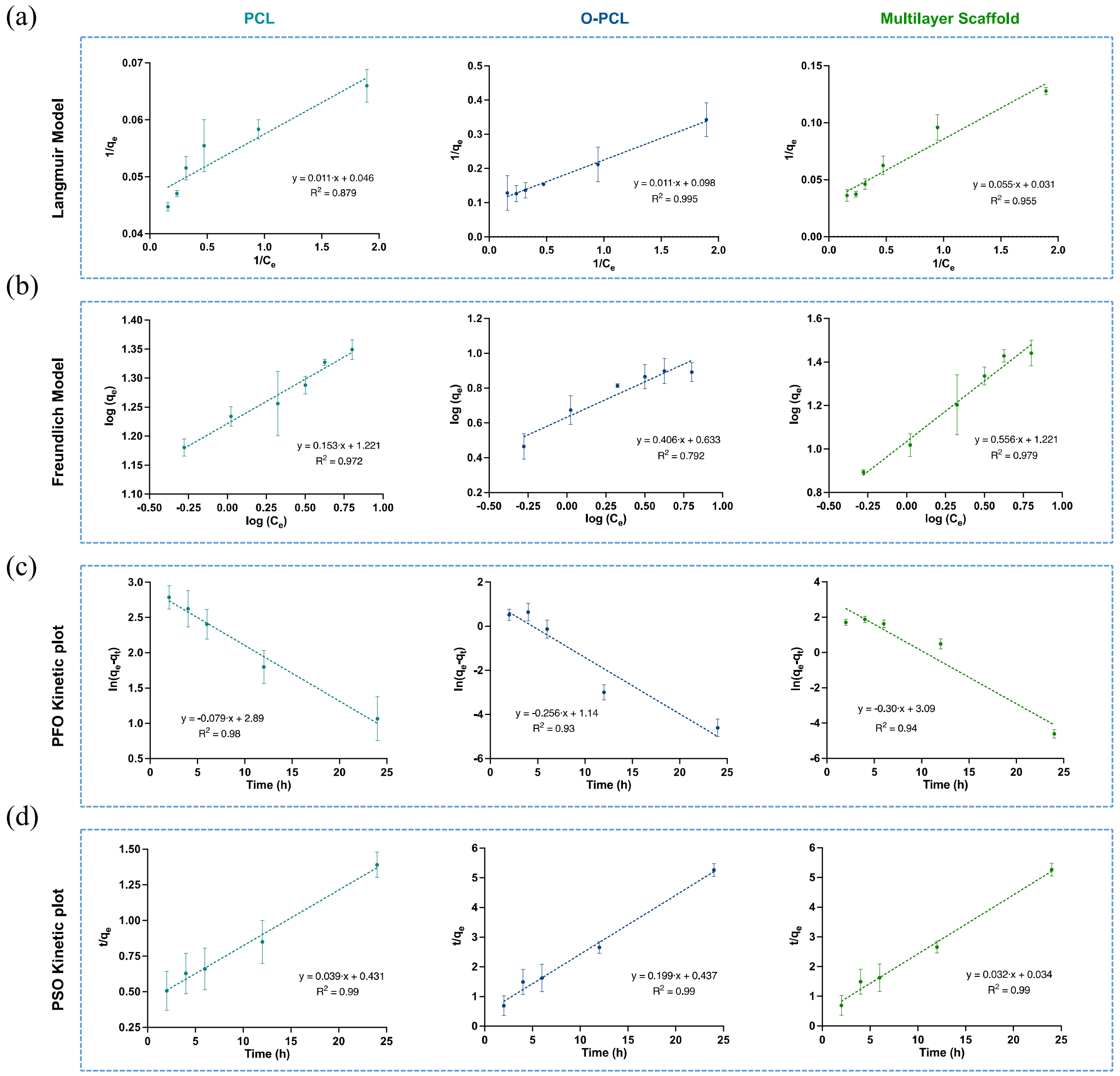

Figure S8. Linearized (a) Langmuir and (b) Freundlich adsorption isotherms at equilibrium. O-PCL and the multilayer scaffold adsorption is likely to proceed by multilayer deposition, while PCL protein adsorption by a monolayer deposition as estimated from the best-fit model. (c) Pseudo-first-order (PFO) and (d) Pseudo-second-order (PSO) kinetic plots for FBS protein adsorption.

References

41. Mohammed, Z.; Jeelani, S.; Rangari, V. Effect of Low-Temperature Plasma Treatment on Surface Modification of Poly-caprolactone Pellets and Thermal Properties of Extruded Filaments. JOM 2020, 72, 1523–1532. https://doi.org/10.1007/s11837-020-04004-y.

77. H. Jeon, H. Lee, and G. Kim, "A Surface-Modified Poly(ɛ-caprolactone) Scaffold Comprising Variable Na-nosized Surface-Roughness Using a Plasma Treatment," Tissue Engineering Part C: Methods, vol. 20, no. 12, pp. 951–963, Dec. 2014, doi: 10.1089/ten.tec.2013.0701.

78. K. Sownthari and S. A. Suthanthiraraj, "Synthesis and characterization of an electrolyte system based on a bio-degradable polymer," Express Polymer Letters, vol. 7, no. 6, pp. 495–504, 2013, doi: 10.3144/expresspolymlett.2013.46.

79. E. Kmiec, Borjigin, Eskridge, Niamat, B. Strouse, and Bialk, “Electrospun fiber membranes enable proliferation of genetically modified cells,” International Journal of Nanomedicine, p. 855, Feb. 2013, doi: 10.2147/IJN.S40117.
80. S. E. Doyle et al., “Characterization of Polycaprolactone Nanohydroxyapatite Composites with Tunable Degra-dability Suitable for Indirect Printing,” Polymers (Basel), vol. 13, no. 2, p. 295, Jan. 2021, doi: 10.3390/polym13020295.
